# Multilayer regulation of CRISPR systems: integrating anti-CRISPR proteins, CRISPRi/a, and quorum sensing networks

**DOI:** 10.3389/fcimb.2026.1844116

**Published:** 2026-05-26

**Authors:** Chunhui Shan, Cong Liu, Chengqiang Jin, Dongxing Tian

**Affiliations:** 1 College of Medical Imaging and Laboratory, Jining Medical University, Jining, Shandong, China; 2 Department of Clinical Laboratory, Affiliated Hospital of Jining Medical University, Jining, Shandong, China; 3 Shandong Key Laboratory of Multi-disciplinary Molecular Diagnosis Precision Medicine, Jining, Shandong, China; 4 Institute of traditional Chinese medicine, Postdoctoral Mobile Station of Shandong University of Traditional Chinese Medicine, Jinan, Shandong, China; 5 State Key Laboratory of Chemical Biology and Drug Discovery and the Department of Food Science and Nutrition, The Hong Kong Polytechnic University, Kowloon, Hong Kong SAR, China

**Keywords:** CRISPR–Cas systems, Anti-CRISPR proteins, CRISPR interference and activation, quorum sensing, multilayer gene regulation, synthetic biology

## Abstract

The CRISPR-Cas system has evolved into a highly efficient platform for genome editing and programmable gene regulation, demonstrating broad application potential in microbiology, biotechnology, and medicine. However, traditional CRISPR tools typically rely on constitutively active nuclease activity; this constant activation state is prone to off-target effects and cytotoxicity, and lacks precise spatiotemporal regulation in complex biological environments. Therefore, developing strategies to achieve fine-tuned and context-dependent regulation of CRISPR activity has become a critical issue in this field that urgently needs to be addressed. Recent studies have demonstrated that various endogenous and exogenous regulatory modules can modulate the activity of the CRISPR-Cas system at different biological levels. Among these, anti-CRISPR proteins (Acr), which are natural inhibitory factors derived from bacteriophages, can suppress the nuclease activity of Cas proteins at the protein level by directly interfering with their function; The CRISPR interference/activation (CRISPRi/a) system, on the other hand, relies on catalytically inactivated Cas proteins to achieve sequence-specific regulation of target gene transcription; furthermore, quorum sensing (QS) networks dynamically regulate the expression of relevant genes by sensing cell density and environmental signals, thereby influencing the functional state of the CRISPR system at the population level. Based on the aforementioned regulatory mechanisms, this paper provides a comprehensive, literature-based overview of the molecular basis and recent advances in the applications of Acr proteins, the CRISPRi/a system, and QS networks in CRISPR-Cas regulation. Building on this, we propose a hierarchical regulatory framework: QS networks serve as upstream environmental sensing modules that drive CRISPRi/a-mediated programmable transcriptional regulation, while Acr proteins act as downstream rapid-response elements that finely tune CRISPR activity. This multi-tiered regulatory system holds promise for the dynamic optimization and precise control of CRISPR systems, offering new design concepts for constructing adaptive, programmable genetic regulatory networks, and demonstrating significant application potential in fields such as microbial engineering, anti-infective strategies, and precision gene regulation. The regulatory mechanism of Acr proteins on CRISPR activity has been experimentally validated in several studies. Nevertheless, the integration of Acr proteins with other regulatory modules such as CRISPRi/a systems or QS networks remains in the exploratory stage and requires further empirical research to confirm their functionality in complex biological systems.

## 1 Introduction

Precision, dynamic, and programmable gene regulation is a key objective of modern biomedical and synthetic biology research. As our understanding of disease mechanisms deepens and the demand for engineering complex biological systems continues to grow, researchers increasingly require genetic tools capable of achieving fine-tuned regulation across different temporal and spatial scales. The discovery and development of the CRISPR-Cas (Clustered Regularly Interspaced Short Palindromic Repeats-Cas) system has provided a key technological platform for this purpose. CRISPR, initially identified in bacterial and archaeal genomes, is an adaptive immune system responsible for defending the host against phage infections and the invasion of exogenous genetic elements (Egido et al., 2022). In this system, CRISPR RNA (crRNA) guides Cas proteins (such as Cas9) to recognize and cleave specific nucleic acid sequences, enabling sequence-specific genome editing. As a result, the engineered CRISPR-Cas system has rapidly emerged as one of the most influential molecular tools in functional genomics, gene therapy, and biotechnology (Bhatia et al., 2023).

Although CRISPR–Cas technology offers highly efficient and specific gene editing capabilities, conventional systems typically rely on the sustained activation of the nuclease function. This “constitutive activity” may lead to off-target effects, cytotoxicity, and additional metabolic burdens in complex biological environments, thereby limiting the safety and controllability of its *in vivo* applications. Consequently, developing strategies to enable fine-tuned and condition-dependent regulation of CRISPR activity has become a key focus of current research. During the long-term co-evolution of bacteria and bacteriophages, a class of natural inhibitory factors derived from bacteriophages—anti-CRISPR proteins (Acr)—has been successively identified (Song et al., 2022). Acr proteins can rapidly block CRISPR system function at the protein level by interfering with the binding of Cas proteins to target DNA or by directly inhibiting their nuclease activity, thereby enabling reversible regulation of the immune response (Zhang and Zhang, 2024). These molecules provide essential tools for constructing CRISPR regulatory systems with switch-like characteristics.

At the same time, by catalytically inactivating Cas proteins (dead Cas, dCas) and fusing them with transcription regulators, researchers have developed CRISPR interference (CRISPRi) and CRISPR activation (CRISPRa) systems (Bendixen et al., 2023). These systems enable reversible regulation of gene expression by sequence-specifically targeting and modulating gene transcription without inducing double-strand breaks in DNA. Consequently, CRISPRi/a has been widely applied in functional genomics research, metabolic engineering, and the development of disease models. Furthermore, by coupling with epigenetic regulatory elements, CRISPRi/a systems can achieve more stable and long-lasting regulation of gene expression (Roth et al., 2024).

However, in complex biological systems, single-level regulatory strategies often fail to achieve precise and dynamic control over CRISPR activity. On the one hand, the regulatory efficiency of CRISPRi/a systems may be limited by factors such as cellular resource allocation, protein expression levels, and the concentration of regulatory elements; on the other hand, in multicellular or microbial community environments, cellular behavior typically relies on coordinated regulation at the population level. Quorum sensing (QS) is a cell-to-cell communication mechanism widely found in bacteria. It detects cell density by secreting and sensing diffusible signaling molecules, thereby coordinating behavioral regulation at the population level, such as biofilm formation, virulence expression, and metabolic activity (Zeng et al., 2023; Guo et al., 2025). Consequently, QS systems provide a natural regulatory layer for constructing regulatory networks capable of responding to environmental changes and enabling collective decision-making. Building on these research advances, recent studies have begun to explore the functional coupling of different regulatory modules—such as Acr proteins, CRISPRi/a systems, and QS networks—to construct genetic regulatory systems with greater adaptability and programmability.

In this review, we provide a conceptual and literature-driven overview of the molecular mechanisms and functional characteristics of Acr proteins, CRISPRi/a systems, and QS networks in CRISPR-Cas regulation, and discuss potential combination strategies for these modules in synthetic biology. Based on these findings, we propose a conceptual multi-tiered regulatory framework: QS networks serve as upstream environmental sensing modules to potentially drive system responses; CRISPRi/a acts as the core transcriptional regulation layer to enable programmable gene regulation; and Acr proteins are envisioned as downstream rapid-response elements to fine-tune CRISPR activity at the protein level. While this hierarchical regulatory system offers a promising approach for dynamic optimization and precise control of the CRISPR system, it is still in the early stages of development and requires extensive experimental validation to confirm its effectiveness.

## 2 Regulatory architecture of the CRISPR–Cas system

### 2.1 Constituent components and functional modules

**Figure 1:**
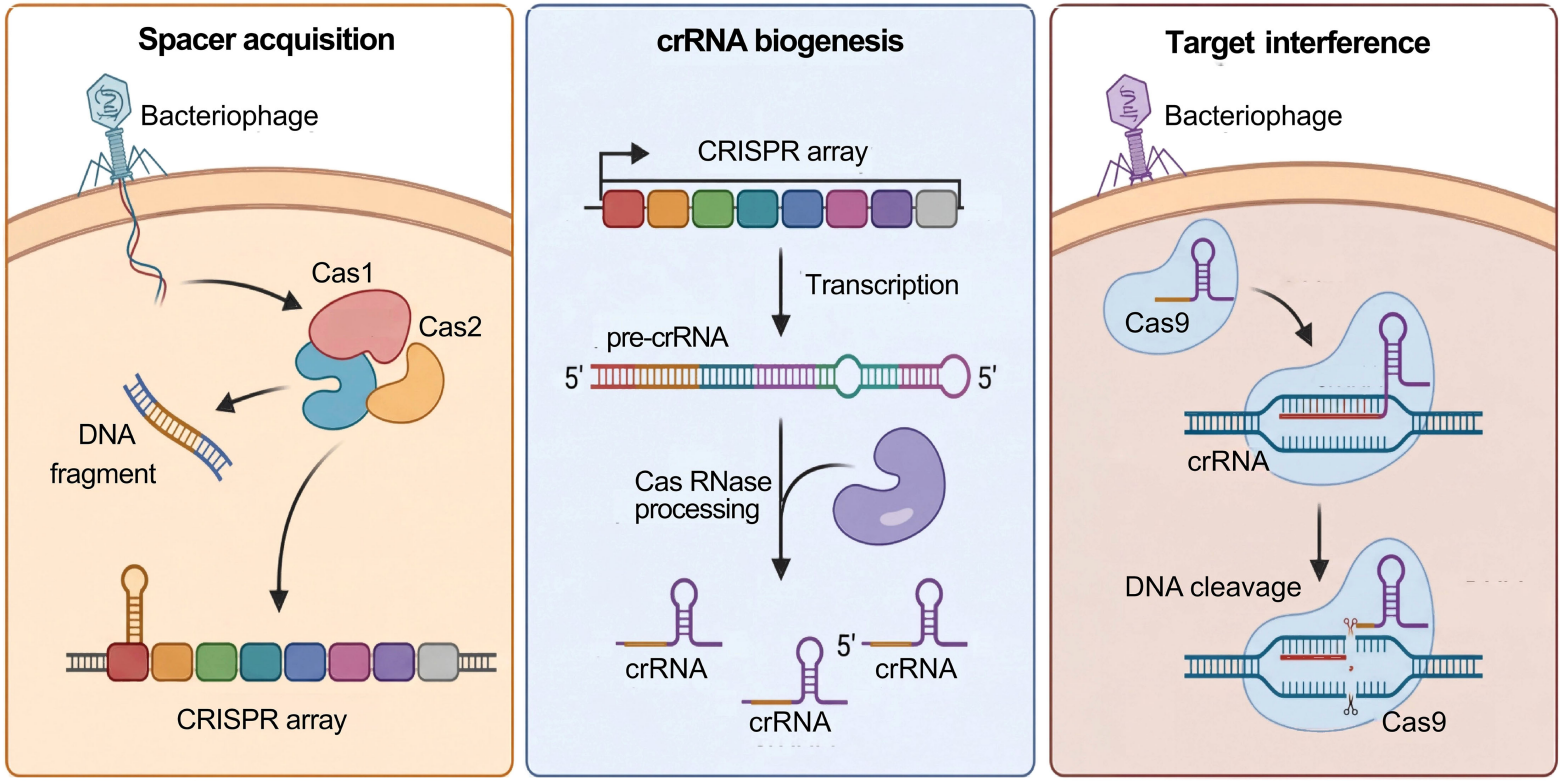
Functional architecture of the CRISPR–Cas immune system. CRISPR–Cas systems provide adaptive immunity in bacteria and archaea against invading genetic elements such as bacteriophages and plasmids. The defense process consists of three major stages. During spacer acquisition, fragments of foreign DNA are captured and integrated into the CRISPR array. In the expression stage, the CRISPR array is transcribed and processed into mature CRISPR RNAs (crRNAs). In the interference stage, crRNAs guide Cas effector proteins to complementary target sequences, leading to sequence-specific cleavage of invading nucleic acids.

The CRISPR–Cas system is an adaptive immune system widely found in bacteria and archaea, serving to defend against the invasion of bacteriophages and other mobile genetic elements (Mitić et al., 2023). This system typically consists of two core genetic modules: the CRISPR array and the adjacent CRISPR-associated (Cas) genes. The CRISPR array forms a “molecular memory” through alternating conserved repeat sequences and spacer sequences derived from exogenous nucleic acids, while the proteins encoded by Cas genes mediate key functions such as target recognition, nucleic acid processing, and cleavage (Hu et al., 2021; Zakrzewska and Burmistrz, 2023) At the functional level, its immune process can be divided into three consecutive stages(**Figure 1**): the adaptation stage (during which the Cas1-Cas2 complex integrates exogenous DNA fragments into the CRISPR array to update immune memory), the expression and processing stage (during which the CRISPR array is transcribed into precursor crRNA and processed into mature crRNA), and the interference stage (during which the mature crRNA guides the Cas effector complex to cleave target nucleic acids) (Gasiunas et al., 2014; Wimmer and Beisel, 2019). This process constitutes the complete functional chain of the CRISPR system, from information acquisition to the execution of defense.

Based on differences in the composition and mechanisms of action of their effector complexes, CRISPR–Cas systems can be classified into several types. Type I systems (such as I-E and I-F) rely on an effector complex composed of multiple protein subunits (the CRISPR-associated complex for antiviral defense, or Cascade) for target recognition and recruit the nuclease Cas3 to degrade the target DNA (Li et al., 2024). In contrast, Type V systems (such as Cas12a) typically rely on a single multifunctional effector protein to perform both target recognition and cleavage (Sinan et al., 2023); Type II systems, on the other hand, are centered around Cas9, requiring only Cas9 and guide RNA (gRNA) to form a sequence-specific effector complex. Due to their relatively simple structure and ease of programming, Type II systems—particularly Cas9—have become the most widely used tools in genome engineering today (Janik et al., 2020).

Based on the aforementioned RNA-guided sequence-specific recognition mechanism, the CRISPR–Cas system has been extensively engineered for gene editing and regulation (Savić and Schwank, 2016). Taking Cas9 as an example, the double-strand breaks it mediates can trigger endogenous DNA repair pathways within cells, thereby enabling different types of genetic modifications: non-homologous end joining (NHEJ) typically results in insertion or deletion mutations, leading to gene knockout (Dalvie et al., 2022); whereas homology-directed repair (HDR) can achieve precise sequence insertion or mutation repair in the presence of a donor template (Zhou et al., 2025). Consequently, CRISPR technology has been widely applied in fields such as disease model construction, functional gene screening, and genetic disease research.

Overall, the CRISPR–Cas system, with its highly modular structure and programmable recognition capabilities, not only forms a crucial foundation for the immune defense of prokaryotes but also provides a key molecular framework for the subsequent construction of multi-level regulatory networks (such as transcriptional and protein-level regulation). This “modular-staged” system characteristic makes it an ideal platform for integrating multiple regulatory elements to achieve fine-tuned control.

### 2.2 Regulatory hierarchies of the CRISPR system

Although the CRISPR–Cas system possesses highly specific and efficient immune functions, its activity must be strictly regulated to prevent potential self-damage to the host genome. Therefore, in natural environments, the CRISPR system is typically embedded within a multi-tiered regulatory network, achieving a dynamic balance between immune defense and cellular homeostasis through fine-tuned regulation of different stages and molecular processes. In terms of regulatory scale, these mechanisms can be broadly categorized into three levels: the transcriptional level, the protein level, and the population level.

At the transcriptional level, host cells regulate the expression of Cas genes and CRISPR arrays through various transcription factors and global regulatory networks. For example, environmentally responsive regulatory factors can upregulate the transcription of CRISPR-associated genes under stress conditions or when the risk of phage infection increases, thereby enhancing immune defense (Agari et al., 2010); conversely, under resource-limited conditions or when immune pressure is low, the expression of these genes can be downregulated to reduce the metabolic burden. Furthermore, the processing efficiency and stability of CRISPR RNA (crRNA) also significantly influence system activity, thereby further refining the regulatory process at the post-transcriptional level.

At the protein level, anti-CRISPR proteins (Acr) constitute a class of key negative regulators. These proteins are typically encoded by bacteriophages or mobile genetic elements and rapidly suppress the immune function of the CRISPR system by directly binding to Cas proteins or effector complexes, thereby interfering with their recognition, binding, or cleavage of target DNA (Marino et al., 2020). This mode of action makes Acr proteins a crucial molecular basis for the immediate regulation and reversible control of CRISPR activity.

In addition to molecular-level regulation, intercellular communication mechanisms also play a crucial role in the regulation of the CRISPR system. Quorum sensing (QS) systems regulate the expression levels of relevant genes by detecting changes in cell density and environmental signaling molecules, thereby coordinating the activation of CRISPR immune responses at the population level (Patterson et al., 2016; Li et al., 2025). This population-based regulatory model enables cells to dynamically adjust their immune strategies in response to the ecological environment, such as enhancing defensive capabilities under high-density conditions or when the risk of infection increases.

In an engineering context, the regulatory hierarchy of the CRISPR system has been further expanded. The CRISPR interference/activation (CRISPRi/a) system, based on catalytically inactivated Cas proteins (dead Cas, dCas), achieves sequence-specific regulation of target gene expression by fusing with transcriptional regulatory domains (Chapman et al., 2023; Srinivasa and Escobar, 2025). This strategy expands the CRISPR platform from a “nucleic acid cleavage tool” to a “programmable transcriptional regulatory module,” providing a critical interface for the construction of multi-level regulatory networks.

In summary, the function of the CRISPR–Cas system is not determined by a single component but rather by a complex network formed through the synergistic interaction of multiple regulatory levels. From transcriptional regulation to protein inhibition and quorum sensing, these mechanisms act in concert across different temporal and spatial scales to achieve fine-tuning and dynamic optimization of CRISPR activity. Notably, this multilevel regulatory architecture not only reflects the complexity of immune defense in natural systems but also provides a theoretical foundation for the subsequent integration of Acr proteins, CRISPRi/a, and QS modules to construct artificial regulatory networks.

## 3 Anti-CRISPR proteins: molecular inhibitors of CRISPR systems

### 3.1 Discovery and classification of anti-CRISPR proteins

Anti-CRISPR proteins (Acr) are a class of natural inhibitory factors encoded by bacteriophages and other mobile genetic elements that promote the replication and spread of exogenous genetic elements by interfering with the host’s CRISPR-Cas immune system. Acr proteins were first identified in 2013 in a bacteriophage infecting Pseudomonas aeruginosa (Bondy-Denomy et al., 2013), revealing for the first time the molecular mechanism by which bacteriophages evade the CRISPR immune system and driving systematic research into the “arms race” between hosts and viruses (Sontheimer and Davidson, 2017) (**Table 1**).

**Table 1:** Summary of anti-CRISPR (Acr) protein families.

Acr family	Target CRISPR type	Mechanism of action	Structural features	Relevant studies
AcrIF	Type I	Interferes with Cascade complex	Small protein (50–150 amino acids), binds to Cas proteins	AcrIF and AcrIE family studies, Pseudomonas aeruginosa
AcrIE	Type I	Inhibits Cas3 nuclease activity	Interferes with Cas3 recruitment, small protein (100 amino acids)	AcrIE and AcrIF inhibition mechanisms, Cryo-EM studies
AcrIIA	Type II	Inhibits Cas9 DNA binding	Small, typically 50–100 amino acids, binds to Cas9	AcrIIA family inhibition of Cas9, structural studies of Cas9 interactions
AcrVA	Type V	Inhibits Cas12a nuclease activity	Directly acetylates Cas12a, contains catalytic domain	AcrVA family studies, Cas12a acetylation, enzymatic inhibition
AcrIIC	Type I	Inhibits Cas3 nuclease activity	Binds to Cascade complex	Cascade binding, AcrIIC family structural studies
AcrIIA2	Type II	Prevents Cas9-sgRNA complex formation	Prevents Cas9 binding to guide RNA	AcrIIA2 interference with Cas9-sgRNA, Neisseria meningitidis
AcrIIC5	Type II	Blocks DNA binding interface of Cas9	Binds to key regions of Cas9, blocking DNA recognition	AcrIIC5 studies on DNA binding, target DNA interference
AcrVA5	Type V	Acetylates key lysine residues on Cas12a	Contains acetyltransferase domain, targets Cas12a	AcrVA5 acetylation of Cas12a, structural studies

To date, through high-throughput genomic mining and functional screening, over 160 Acr protein families have been identified, widely distributed among phages, plasmids, and integrative mobile genetic elements (Khatri et al., 2025). Despite their high sequence diversity, Acr proteins typically have a small molecular weight (approximately 50–150 amino acids) and efficiently disrupt key functional steps of the CRISPR system through specific protein-protein interactions or enzymatic modifications.

Based on the type of CRISPR system they target, Acr proteins can be classified into different functional categories. For example, the AcrIF and AcrIE families specifically inhibit Type I CRISPR systems by interfering with the multi-subunit effector complex; the AcrIIA family targets Type II systems, while the AcrVA family acts on Type V systems, directly inhibiting individual effector proteins (such as Cas9 or Cas12) (Yu and Marchisio, 2020; Wang et al., 2024). This “target type”-based classification reflects the selective adaptation of Acr proteins to different CRISPR systems. Notably, even when targeting the same system type, different Acr families exhibit significant differences in their molecular inhibition mechanisms, reflecting the high degree of functional differentiation that has emerged during their coevolution. Overall, the diversity of Acr proteins in terms of origin, structure, and mechanism of action makes them a crucial entry point for elucidating the regulatory complexity of CRISPR systems.

### 3.2 Molecular inhibition mechanisms of anti-CRISPR proteins

**Figure 2:**
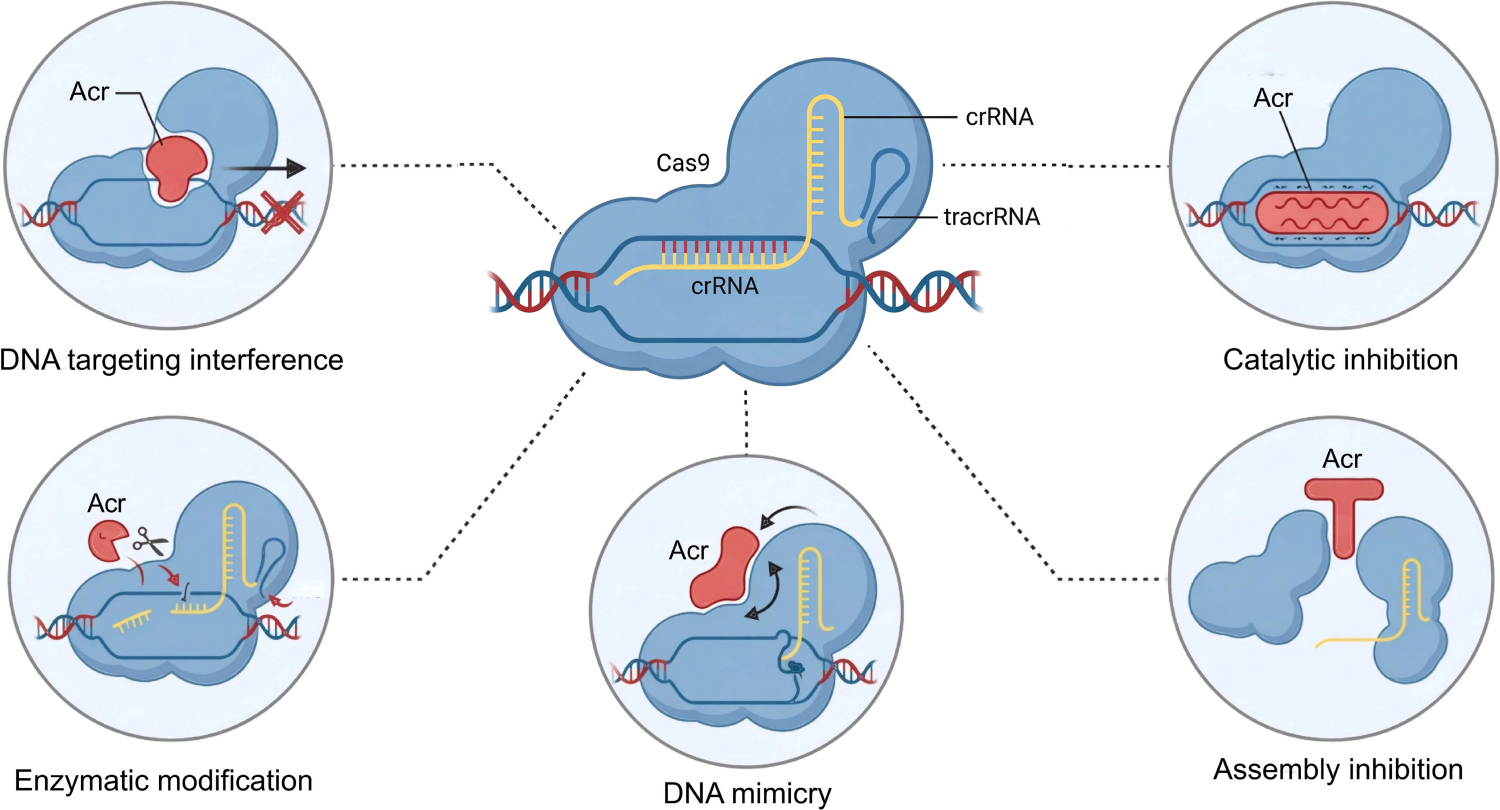
Molecular mechanisms of anti-CRISPR protein–mediated inhibition. Anti-CRISPR proteins are natural inhibitors evolved by bacteriophages to counteract bacterial CRISPR immunity. These proteins suppress CRISPR activity through multiple mechanisms, including blocking Cas protein binding to target DNA, inhibiting nuclease activity, or preventing the assembly of CRISPR effector complexes. Different anti-CRISPR families display diverse structural strategies to achieve efficient inhibition of CRISPR systems.

To counter the CRISPR-Cas immune system, bacteriophages have evolved a diverse arsenal of anti-CRISPR proteins (Acr proteins) (Choudhary et al., 2023). These proteins efficiently inhibit the host defense system at nearly every critical step through various molecular mechanisms. Based on their targets and modes of action, the inhibition strategies of Acr proteins can be classified into the following major categories (**Figure 2**).

The first category of mechanisms acts by interfering with the assembly of the CRISPR effector complex or the loading of guide RNA (Semenova et al., 2011). In the CRISPR-Cas system, Cas proteins must first form a ribonucleoprotein (RNP) complex with crRNA (or sgRNA), which serves as the foundation for subsequent target recognition. Therefore, blocking this early step can inhibit the activation of the CRISPR immune response at its source. For example, AcrIIC2 binds to the positively charged bridge-helix region of Neisseria meningitidis Cas9 (Nme1Cas9) in a dimeric form, thereby interfering with guide RNA binding (Thavalingam et al., 2019). Since Cas9 cannot acquire guide RNA, it completely loses its ability to recognize target DNA, and the immune response is terminated at the very earliest stage.

The second class of mechanisms acts by preventing the CRISPR effector complex from recognizing or binding to target DNA (Sun et al., 2023a). Target recognition by the CRISPR system relies on PAM sequence recognition followed by base pairing between the guide RNA and the target DNA; various Acr proteins exploit this critical step to interfere with the process. For example, studies have found that AcrIIC5Nch, an anti-CRISPR protein homolog from Neisseria chenii, blocks the binding of Neisseria meningitidis type II C-type Cas9 (Nme1Cas9) to DNA by targeting its PAM interaction domain (PID). This inhibitory effect depends on the pre-loading of guide RNA onto Cas9, and its mechanism of action is similar to that of the AcrIIA family of proteins (Hwang et al., 2023); AcrIIC4 inhibits double-stranded DNA unwinding by stabilizing the cleft between the REC1 and REC2 domains of Cas9, thereby preventing R-loop formation and blocking the binding of the effector complex to target DNA. Other Acr proteins may bind to critical structural regions of Cas proteins, directly blocking the DNA-binding interface. Through such mechanisms, the CRISPR system is effectively neutralized before DNA cleavage occurs.

The third class of inhibitory mechanisms targets the catalytic activity of Cas proteins, acting during the DNA cleavage phase. Even if the CRISPR effector complex has successfully bound to the target DNA, its double-strand cleavage activity still depends on the conformational activation of the nuclease domain. Some Acr proteins directly block this critical step by specifically binding to the catalytic domain or by stabilizing the enzyme in its inactive conformation. Taking AcrIIC1 as an example, this protein binds to the HNH nuclease domain of Neisseria meningitidis Cas9, inhibiting its rearrangement into the catalytic conformation and thereby effectively suppressing DNA cleavage activity. However, this process does not affect the recognition and binding of the Cas9-sgRNA complex to the target DNA (Trasanidou et al., 2023). Under such mechanisms, although the CRISPR complex retains its full DNA-binding capacity, its nuclease function is specifically inactivated during the execution phase, demonstrating the precise targeting of Acr proteins toward functional nodes of the CRISPR system.

Another unique and highly efficient inhibition mechanism involves enzymatic modification or degradation of key molecular components. These Acr proteins possess catalytic activity themselves and can induce irreversible changes in key molecules. Studies have shown that AcrVA5 directly inhibits the nuclease activity of Cas12a by acetylating specific lysine residues (Dong et al., 2019; Kang et al., 2022). Compared to other inhibition methods, this mechanism achieves a more persistent and even irreversible inhibitory effect through direct chemical modification.

A final, distinct inhibition strategy involves DNA mimicry (Kang et al., 2023). Studies reveal that AcrIIC5 mimics the structure and charge distribution of double-stranded DNA; its negatively charged groove specifically binds to and occupies the PAM recognition site of the Cas9 protein, thereby blocking target DNA binding and achieving highly efficient inhibition of Cas9 type II-C (Sun et al., 2023b). Through this “molecular camouflage” strategy, Acr proteins can achieve highly efficient inhibition without directly altering the conformation of the Cas protein.

### 3.3 Inhibition mechanisms of different types of CRISPR–Cas systems

Although anti-CRISPR proteins share certain commonalities in their molecular mechanisms, their specific modes of action exhibit marked differences across different types of CRISPR–Cas systems. These differences primarily stem from variations in the structural composition, form of the effector complex, and target recognition mechanisms of each system, resulting in significant differences in their sensitivity to various inhibition strategies.

#### 3.3.1 Type I CRISPR–Cas system

The Type I CRISPR–Cas system relies on the Cascade (CRISPR-associated complex for antiviral defense, or Csy) effector complex—composed of multiple protein subunits—to recognize target DNA and recruit the nuclease Cas3 to mediate the processive degradation of the target DNA (Kim et al., 2024). Characterized by “multi-subunit recognition + single-nuclease execution,” this system’s functional process involves a complex network of protein–protein and protein–DNA interactions, thereby exposing potential regulatory and intervention sites at multiple stages. Consequently, anti-CRISPR proteins targeting the Type I system (primarily from the AcrIF and AcrIE families) typically exhibit preferential inhibition of the target recognition and effector complex functional coupling processes.

In terms of molecular mechanisms, inhibition of Type I systems primarily focuses on the following key steps. First, during the target DNA recognition stage, some Acr proteins can directly bind to key subunits of the Cascade complex (such as Cas7 or Cas8), blocking their recognition of the PAM sequence or interfering with base pairing between crRNA and target DNA, thereby inhibiting the DNA-targeting process. Second, some Acr proteins interfere with the interaction between the Cascade complex and Cas3, blocking the recruitment of Cas3 or inhibiting its nuclease activity. This causes the system to remain in an intermediate “recognized but not executed” state, resulting in functional decoupling from the effector phase (Zhang et al., 2019; Kang et al., 2025). Furthermore, certain Acr proteins can induce the Cascade complex to bind to non-specific DNA, altering its spatial distribution within the cell, thereby reducing its target-search efficiency and generating an “exhaustion effect” (Lu et al., 2021).

Overall, inhibition strategies for Type I CRISPR–Cas systems primarily target the recognition interface of the effector complex and its downstream functional coupling mechanisms. This inhibition logic, centered on “structural exposure,” highlights the susceptibility of multimeric systems to structural interference and provides a crucial reference for understanding the differences in regulatory mechanisms between them and monomeric systems.

#### 3.3.2 Type II CRISPR–Cas system (Cas9)

The Type II CRISPR–Cas system centers on a single effector protein, Cas9, which forms a ribonucleoprotein complex by binding to single-guide RNA (sgRNA), thereby enabling the recognition and cleavage of target DNA (Wang et al., 2016). Unlike Type I systems, which rely on multi-subunit complexes, Cas9 integrates functions such as RNA binding, PAM recognition, and nucleic acid cleavage into a single molecule, while structurally exhibiting functional compartments composed of the recognition domain (REC) and the nuclease domains (HNH and RuvC) (Zhang et al., 2025). This characteristic of “single-molecule integration with distinct structural compartments” exposes potential regulatory sites at multiple functional nodes in the Type II system, thereby exhibiting high mechanistic adaptability.

At the molecular level, anti-CRISPR inhibitors targeting Cas9 can act on multiple stages of its functional cycle. First, during the complex assembly stage, certain Acr proteins (such as AcrIIA2) can interfere with the binding of Cas9 to sgRNA or reduce complex stability, thereby inhibiting the formation of functional ribonucleoprotein complexes—a process known as assembly inhibition. Second, during the target recognition stage, AcrIIA13b binds to the PAM recognition interface of Cas9, mimics the PAM sequence, and occupies its critical binding region, thereby blocking the initial binding of Cas9 to the target DNA through steric hindrance. This mechanism is described as “PAM mimicry” and directly interferes with the DNA-targeting process of the CRISPR-Cas9 system (Lee and Park, 2025). Furthermore, during the catalytic activation stage, Cas9’s cleavage activity depends on the precise positioning of the HNH domain toward the target DNA strand and the synergistic action of the RuvC domain. Consequently, certain Acr proteins can achieve allosteric or catalytic inhibition of nuclease activity by stabilizing the inactive conformation of Cas9 or interfering with the dynamic rearrangement between domains (Belato and Lisi, 2023). Notably, some Acr proteins (such as AcrIIA6) can also induce Cas9 dimerization, thereby impairing its normal DNA-binding ability; this mechanism further expands the molecular strategies for regulating Cas9 function (Fuchsbauer et al., 2019). These diverse inhibition patterns indicate that Cas9 not only exposes regulatory windows at multiple functional nodes, but its dynamic conformational changes themselves also constitute important intervention targets.

Overall, inhibition of the Type II CRISPR–Cas system spans the entire functional process from complex assembly and target recognition to nucleic acid cleavage, demonstrating significant “multi-site intervenability.” This characteristic stems from the highly integrated yet dynamically coupled functional modules within the Cas9 monomeric structure, making it more susceptible to fine-tuned regulation at the molecular level. Consequently, Cas9 is not only a key target for natural Acr proteins but has also emerged as one of the most malleable CRISPR effector proteins in engineered regulatory strategies.

In engineering applications, Acr proteins have been widely used to regulate Cas9-mediated gene editing processes. For example, by inducing or tissue-specifically expressing Acr proteins, spatiotemporal-specific inactivation of Cas9 activity can be achieved, thereby effectively reducing off-target effects and enhancing the safety of *in vivo* applications (Nakamura et al., 2019; Zhuo et al., 2021). Furthermore, coupling the Acr module with CRISPRi/a systems or environmental response circuits (such as QS systems) enables the construction of programmable genetic circuits with feedback regulation capabilities, further expanding the application potential of CRISPR systems in synthetic biology (Gao et al., 2026).

#### 3.3.3 Type V CRISPR–Cas system (Cas12)

The Type V CRISPR–Cas system is represented by the Cas12 protein, which is also a single-protein effector; however, its functional mechanism differs significantly from that of Cas9. Cas12 relies on crRNA to form a functional complex and undergoes conformational activation upon recognition of target DNA. It not only mediates the cleavage of target DNA but also triggers non-specific collateral cleavage activity (Saha et al., 2020; Ma et al., 2022). This “activation-dependent” catalytic mode makes it highly dependent on complex integrity and RNA components, thereby shaping its unique inhibition strategies.

At the molecular mechanism level, anti-CRISPR inhibition targeting Cas12 primarily acts on the following key steps. First, regarding complex assembly and stability, inhibitors can disrupt the integrity of the ribonucleoprotein complex by interfering with crRNA binding or reducing its stability, thereby inhibiting functional activation (Knott et al., 2019). Second, during the target recognition stage, Cas12 relies on PAM recognition and the unwinding of double-stranded DNA to initiate the targeted reaction; consequently, its DNA-binding interface can also serve as a target for inhibition (Jeon et al., 2018). More importantly, at the catalytic level, Cas12’s nucleic acid cleavage activity depends on the RuvC domain and its key catalytic residues (Zheng et al., 2025). Consequently, certain Acr proteins (such as AcrVA5) can directly inhibit its nuclease activity by acetylating key lysine residues; this mechanism represents a classic “enzyme-mediated modification-based inhibition strategy.”

Overall, inhibition in Type V systems places greater emphasis on the regulation of “catalytic state and chemical modification” compared to Type II systems. This characteristic stems from the high dependence of Cas12 activity on substrate-induced conformational changes and the chemical state of key residues, making it an important model for studying Acr-mediated “enzyme activity regulation” mechanisms.

## 4 Programmable gene regulation: CRISPRi and CRISPRa

### 4.1 Mechanistic framework of CRISPRi and CRISPRa

**Figure 3:**
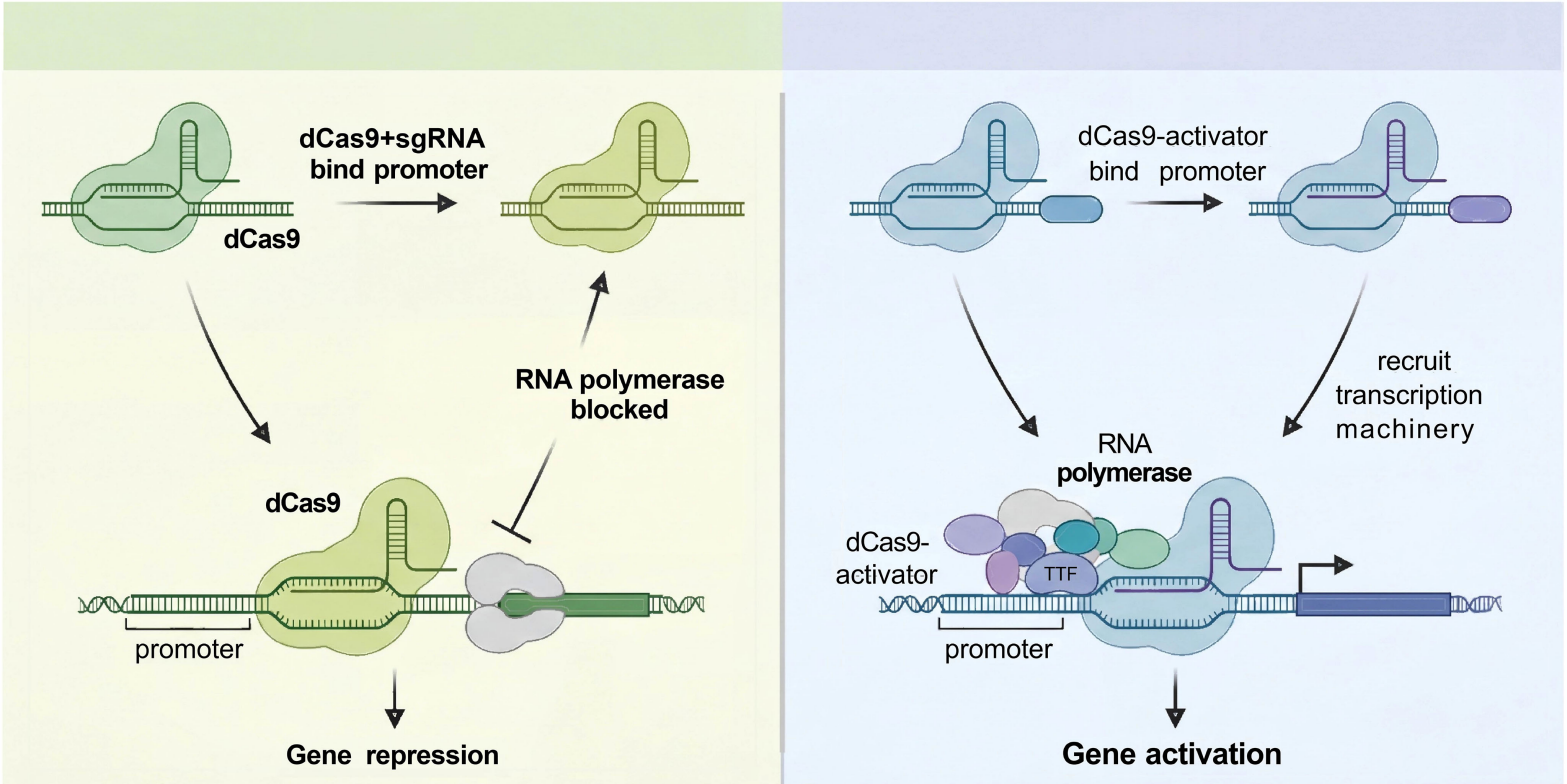
Programmable transcriptional regulation using CRISPR interference (CRISPRi) and CRISPR activation (CRISPRa). Catalytically inactive Cas proteins (dCas) retain their DNA-binding ability but lack nuclease activity. In CRISPRi systems, dCas proteins guided by sgRNAs bind promoter or coding regions and block transcription, leading to gene repression. In CRISPRa systems, dCas proteins fused to transcriptional activators recruit transcription machinery to target promoters, resulting in enhanced gene expression.

The development of CRISPR interference (CRISPRi) and CRISPR activation (CRISPRa) has transformed the CRISPR–Cas system from a simple nucleic acid cleavage tool into a programmable gene regulation platform. At the core of these two technologies are catalytically inactivated Cas proteins (such as dCas9 or dCas12), which retain sequence-specific DNA-binding capabilities but lack nuclease activity. By coupling programmable DNA targeting with modular effector functions, CRISPRi/a systems establish a universal framework for the precise and reversible regulation of gene transcription. (**Figure 3**).

CRISPRi achieves gene silencing by converting sequence-specific DNA binding into transcriptional inhibition. Guided by a single-guide RNA (sgRNA), dCas proteins are recruited to promoter regions or transcriptional sequences, where they interfere with transcription through two primary and synergistic mechanisms. First, the dCas protein acts as a spatial blocker, directly impeding RNA polymerase binding, transcription initiation, or elongation. This inhibition is highly site-dependent, generally yielding the strongest inhibitory effect when targeting regions near the transcription start site (TSS) (Peddle et al., 2020; Vercauteren et al., 2024). Second, fusing the dCas protein with transcription-repressive domains can further enhance the functionality of CRISPRi. In eukaryotic systems, KRAB domains are commonly used to recruit endogenous co-repressor complexes, including HP1 and SETDB1, which catalyze the formation of repressive chromatin modifications (such as H3K9 trimethylation), leading to the formation of compact heterochromatin and the establishment of a more stable, heritable silenced state (Groner et al., 2010; Yan et al., 2023). In prokaryotes, due to the simpler chromatin structure, inhibition is primarily achieved through direct competition with RNA polymerase. These mechanisms make CRISPRi a robust and tunable gene silencing platform, where the intensity and reversibility of repression can be modulated through sgRNA design, target site selection, and the type of effector domain.

In contrast to CRISPRi, the CRISPRa system enhances gene expression by converting DNA targets into the recruitment of local transcriptional activation complexes. When the dCas protein is directed to a region near the promoter, it promotes the assembly or stabilization of the transcriptional machinery, thereby enhancing transcription initiation efficiency. The efficiency of CRISPRa is highly dependent on the positioning of the sgRNA within the regulatory element. Studies have shown that targeting the region approximately −50 to −400 bp upstream of the TSS typically yields the best activation results, although the specific range varies depending on promoter structure and cell type (Horlbeck et al., 2016; Dong et al., 2018). Mechanistically, CRISPRa systems can be classified into three main architectures: First, direct fusion systems (e.g., dCas9–VP64) achieve compact regulation with limited activation potency by covalently linking a single transcriptional activation domain to the dCas protein. The second category, multi-domain fusion systems (e.g., VPR), combines VP64, p65, and Rta activation domains to produce synergistic enhancement effects, achieving higher levels of transcriptional activation (Guo et al., 2017). The third category, modular recruitment systems (e.g., SunTag and SAM), utilizes scaffolding strategies to recruit multiple activator molecules to a single gene site, enabling signal amplification and providing greater regulatory flexibility (Fu et al., 2025).

Although CRISPRi and CRISPRa mediate opposite transcriptional outcomes, they share a core design logic: programmable DNA targeting and modular transcriptional regulation. CRISPRi primarily relies on spatial blocking and epigenetic repression, resulting in robust and predictable gene silencing. CRISPRa relies on the efficient recruitment and coordinated assembly of activation complexes, making it more sensitive to the genomic context of the target gene and design parameters. Furthermore, both systems are highly modular and scalable, enabling the simultaneous regulation of multiple genes through the use of multiple sgRNAs. This lays the foundation for the construction of complex genetic networks, making them suitable for advanced synthetic biology applications.

### 4.2 Applications of CRISPRi/a in synthetic biology and gene regulation

The programmability, reversibility, and scalability of the CRISPRi/a system have led to its widespread use in synthetic biology, making it a core tool for engineering gene expression, metabolic pathways, and regulatory networks.

#### 4.2.1 Metabolic engineering

CRISPRi/a enables precise regulation of metabolic flux without the need for permanent genetic modification. By inhibiting competing pathways (CRISPRi) and activating rate-limiting enzymes (CRISPRa), metabolic resources can be precisely allocated to target products. Compared to traditional gene knockout or overexpression methods, CRISPRi/a allows for graded and combinatorial control of gene expression, making it particularly suitable for optimizing complex biosynthetic pathways. For example, simultaneously downregulating branching enzyme activity via a multi-target CRISPRi system while enhancing the activity of key node enzymes with CRISPRa significantly increases the yield of biofuels, amino acids, and natural products (Das et al., 2024). This dynamic and adjustable pathway control strategy is well-suited for iterative strain optimization and adaptive metabolic engineering.

#### 4.2.2 Genetic circuit engineering

In synthetic genetic circuit design, the CRISPRi/a system can serve as a programmable regulatory module to implement logical operations and dynamic control behaviors. By leveraging the programmable nature of sgRNAs, transcriptional networks that mimic electronic circuits can be constructed, including logic gates (AND, OR, NOT), feedback loops, and oscillators (Santos-Moreno et al., 2020; Jeong et al., 2023). Due to its low leakage and predictability, CRISPRi is suitable for inhibitory circuits, while CRISPRa is suitable for activating networks and signal amplification. The orthogonality of sgRNAs allows multiple regulatory interactions to be encoded within the same cell without interfering with one another (Bervoets and Charlier, 2019; Diandreth et al., 2022; Allemailem et al., 2023). These characteristics enable researchers to develop complex, multilayer regulatory networks and programmable cellular state switches, driving the advancement of biological computing toward scalability and modularity.

#### 4.2.3 Multi-gene and network-level regulation

A major advantage of CRISPRi/a systems is their ability to regulate multiple targets. By co-expressing multiple sgRNAs, dozens of genes can be coordinated and controlled within a single system, enabling network-level regulation (Fang et al., 2021; Enright et al., 2024). This feature is particularly important in functional genomics and systems biology, where it can be used for high-throughput screening, gene function identification, and studies of regulatory dependencies (Jaffe et al., 2019; Karlson et al., 2021). Furthermore, multi-target CRISPRi/a can reconfigure and reprogram endogenous gene networks, aiding in the study of complex traits and cellular decision-making mechanisms. From a systems perspective, CRISPRi/a elevates single-gene perturbation to global regulatory reprogramming, providing a powerful framework for deciphering and engineering biological complexity.

## 5 Quorum sensing as an upstream regulatory layer of the CRISPR system

Quorum sensing (QS) is a regulatory mechanism that enables population density-dependent gene expression through the secretion and detection of small signaling molecules (Grandclément et al., 2016). In many microorganisms, QS not only controls biofilm formation, virulence factors, and metabolic pathways, but also exerts upstream regulation on the CRISPR-Cas system (Qin et al., 2022; Sanchez-Torres et al., 2024). By coupling QS signals with CRISPR function, microorganisms can flexibly modulate their immune responses in response to changes in population density or environmental stress, thereby optimizing resource utilization and enabling dynamic defense.

### 5.1 Molecular mechanisms of QS

QS is a cellular communication mechanism by which bacteria coordinate gene expression by monitoring their own population density. This system typically consists of three components: a signal molecule synthase, a diffusible signal molecule, and a corresponding receptor or transcription factor. Gram-positive bacteria primarily utilize lipopeptide signaling, sensing population density through the secretion of autoinducing peptides (AIPs). Upon binding to extracellular receptors, AIPs activate a two-component signaling system, which in turn regulates downstream target genes (Aboelnaga et al., 2024); Gram-negative bacteria, on the other hand, predominantly employ N-acyl-homoserine lactone (AHL)-type signaling molecules. Because these molecules can freely cross the cell membrane, they bind to LuxR-type receptor proteins to form active complexes once intracellular concentrations accumulate to a threshold, thereby initiating the expression of specific genes (Fray, 2002). At the molecular level, QS signals precisely control the expression of downstream genes by altering the DNA-binding affinity of receptor proteins or recruiting transcription cofactors, thereby enabling bacteria to dynamically adjust their physiological behavior in response to factors such as population density, nutritional status, and environmental stress.

### 5.2 Regulation of the CRISPR system by QS

The regulation of the CRISPR-Cas system by QS exhibits a multi-level nature, reflecting the sophisticated control of immune defense at the population level in bacteria. At the transcriptional level, QS signaling molecules can directly regulate the expression of Cas protein-encoding gene clusters and guide RNAs. For example, in Pseudomonas aeruginosa, the AHL-type QS system regulates the transcriptional activity of the Type I CRISPR-Cas system via the LasR/RhlR transcription factors, thereby influencing the assembly efficiency of the Cascade effector complex and the strain’s resistance to phages (Singh et al., 2015). At the level of immune activity regulation, QS achieves “threshold control” of CRISPR immune strength by coordinating the relative expression levels of Cas proteins and crRNAs (Wang et al., 2023). Specifically, it suppresses immune activity at low population densities to conserve energy, while enhancing defensive capabilities once the population density reaches a certain threshold to counter the risk of phage transmission. Concurrently, QS can form closed-loop feedback circuits with CRISPRi/a gene regulatory systems or Anti-CRISPR (Acr) proteins to achieve dynamic temporal control of the immune response—rising QS signal concentrations trigger Cas protein expression (Du et al., 2021), while Acr or CRISPRi suppress system activity at specific stages, thereby avoiding the adaptive costs associated with immune overactivation. Furthermore, universal signaling molecules such as AI-2 can mediate inter-population communication within mixed bacterial communities, enabling different bacterial species to coordinately regulate CRISPR-Cas activity in the face of a shared phage threat, thereby forming a collective defense strategy (Mayer et al., 2023; Markowska et al., 2024). These findings reveal that QS acts as a central regulator of collective behavior, embedding the CRISPR immune system into the social adaptation network of bacteria through multiple mechanisms.

### 5.3 Applications in synthetic biology

The functional coupling of quorum sensing (QS) with CRISPR systems provides the field of synthetic biology with programmable, dynamic molecular tools capable of population-level regulation, giving rise to a wide range of applications. By leveraging the population density sensing capabilities of QS systems and combining them with the precise gene regulation capabilities of CRISPR, researchers have developed a variety of innovative genetic circuits and regulatory networks.

#### 5.3.1 Population density-responsive gene regulation

By placing CRISPR interference (CRISPRi) or activation (CRISPRa) systems under the transcriptional control of QS signals, population density-dependent gene expression regulation can be achieved. A typical strategy involves placing the expression of guide RNAs (sgRNAs) or inactivated Cas proteins (dCas9) under the control of the LasR-type QS system in Pseudomonas aeruginosa, ensuring that gene silencing or activation is initiated only when signal molecule concentrations reach a threshold (Haval et al., 2025). This strategy demonstrates significant application value in microbial fermentation engineering, as it can be used to dynamically regulate metabolic flux, avoid early metabolic burdens, and thereby improve the yield and conversion efficiency of target products.

#### 5.3.2 Programmable immune and defense networks

QS-regulated CRISPR-Cas systems provide dynamic regulatory capabilities for anti-phage defense in synthetic ecosystems. By designing a QS-signal-controlled CRISPR system, cells can suppress immune activity to conserve energy under conditions of low population density and limited energy resources, while activating defense functions when population density increases and the risk of phage transmission rises (Hobbs and Kranzusch, 2024; Haval et al., 2025). This population-state-based immune trade-off mechanism is of great significance for maintaining stability in microbial co-cultures and synthetic ecosystems.

## 6 Multidimensional integration and rational design of CRISPR regulatory systems

With increasing insights into Acr, CRISPRi/a, and QS mechanisms, CRISPR-based regulation is shifting from single-module use toward multidimensional integration. These modules differ in regulatory level, response speed, reversibility, host range, implementation constraints, and typical applications, collectively shaping their functional roles. Acr proteins act post-translationally by directly inhibiting Cas effectors, enabling rapid and reversible control. CRISPRi/a mediates transcriptional regulation with slower but stable responses, while QS coordinates population-level gene expression through threshold-dependent signaling (Williams and Cámara, 2009; Dominguez et al., 2016; Wang et al., 2019; Liang et al., 2022). These temporal and hierarchical distinctions suggest complementarity, yet systematic cross-layer integration remains limited.

Regarding host range, CRISPRi/a is broadly applicable, Acr is Cas-type dependent, and QS is largely bacterial, with cross-species deployment hindered by signal compatibility. Implementation constraints vary, including metabolic burden and circuit complexity. Typical applications reflect these properties: CRISPRi/a for functional genomics and metabolic engineering, Acr for safety control and “off-switch” functions, and QS for collective behavior and community-level regulation. Most studies focus on single layers or pairwise combinations, and generalizable frameworks are still lacking.

Overall, these modules are functionally complementary (**Table 2**), but integrating them poses challenges in kinetic matching, interoperability, and stability. Current strategies fall into three representative design paradigms, partially validated experimentally but not yet fully generalizable.

**Table 2:** Comparative analysis of regulatory modules:Acr,CRISPRi/a and quorum sensing.

Regulatory level	Response speed	Reversibility	Host range	Implementation constraints	Typical use cases
Acr Proteins	Fast	High	Limited to specific Cas types	Cas-specific, limited cross-platform functionality	Dynamic control of CRISPR systems, safety switches
CRISPRi/a	Slow	Medium	Broad (Prokaryotes and Eukaryotes)	Requires transcriptional control systems, slower regulation	Gene regulation, metabolic engineering, functional genomics
Quorum Sensing	Medium	Low	Mainly bacteria	Signal diffusion, background noise, cellular heterogeneity	Population-level regulation, biofilm formation, virulence expression

### 6.1 Protein-level dynamic regulatory system based on Acr–CRISPRi/a coupling

By specifically binding to the Cas complex, the Acr protein provides an orthogonal protein-level inhibition mechanism for the CRISPRi/a system, thereby enabling a dual-layer regulatory strategy that integrates post-translational control with transcriptional regulation (Belato and Lisi, 2023). This dynamic regulatory system typically comprises two core modules: first, the transcriptional effector module (dCas9-effector protein/sgRNA), responsible for target gene regulation; and second, the signal-responsive control module (the Acr gene driven by an inducible promoter), used to achieve exogenous regulation of the system. The core of this system lies not in simple “on/off” switching, but in a reversible regulatory process based on protein interaction kinetics (Dong et al., 2017). Under non-induced conditions, Acr expression remains at low levels, allowing the dCas9 complex to function normally (Kim et al., 2018). Upon introduction of an exogenous inducer, Acr protein is rapidly expressed and inactivates the dCas9–sgRNA complex through steric hindrance or allosteric effects, thereby achieving immediate suppression of the system’s function (Pawluk et al., 2018). Upon removal of the signal, system recovery depends on the degradation kinetics of intracellular Acr and the reassembly capacity of the dCas9 complex. Compared to traditional transcriptional regulation, this design significantly reduces response latency, enabling high-resolution temporal control over gene editing or regulatory windows (Del Vecchio et al., 2016). Notably, such systems are expected to exhibit asymmetric dynamic behavior, characterized by rapid inhibition and comparatively slower recovery, which may provide advantages for fine-tuned and time-resolved regulation. However, current implementations are largely limited to engineered constructs or proof-of-concept studies, and their kinetic properties, as well as stability across different host organisms, remain to be systematically evaluated.

### 6.2 Integration of QS and CRISPR systems: environmentally responsive genetic circuits

The integration of the CRISPR system with quorum sensing (QS) transforms gene regulation from a static expression pattern into a dynamic, environment-responsive system. Within this framework, the QS module (such as the LuxI/LuxR-AHL system) serves as the sensing layer, converting cell density information into a regulatable molecular signal; the CRISPRi/a module (dCas9 effector and sgRNA) serves as the execution layer, enabling precise regulation of target genes. Depending on the coupling mechanism, such systems typically exhibit two distinct regulatory modes: First is the density-dependent activation mode. When cell density increases, causing AHL signaling to accumulate beyond a threshold, the CRISPRi/a system is induced, thereby regulating the target gene. This mode enables population-scale-dependent gene expression regulation. Second is the self-regulatory feedback mode. At low densities, the CRISPRi/a system remains active; as population density increases, QS signals trigger inhibitory pathways (e.g., Acr expression), forming a negative feedback regulatory loop (Ghosh et al., 2022). This mechanism helps prevent system overactivation and enhances regulatory stability. This modular design enables engineered cells to sense microbial signals across temporal and spatial dimensions and dynamically adjust gene expression and metabolic flux, thereby achieving programmatic and logical regulatory behaviors. Currently, the coupling of quorum sensing (QS) and CRISPR systems has been validated in various synthetic microorganisms. A related study introduced a tool called the “QS-controlled type I CRISPR interference (QICi) toolkit,” which uses quorum sensing signals to regulate CRISPR interference and dynamically modulate metabolic pathways (Zheng et al., 2025). This approach has successfully achieved metabolic rewiring in *Escherichia coli*, significantly improving biomanufacturing efficiency. However, the stability, signal specificity, and resistance to interference of this system in complex environments remain critical challenges that need to be addressed.

### 6.3 Construction of a hierarchical CRISPR regulatory network: integrating Acr, CRISPRi/a, and QS

**Figure 4:**
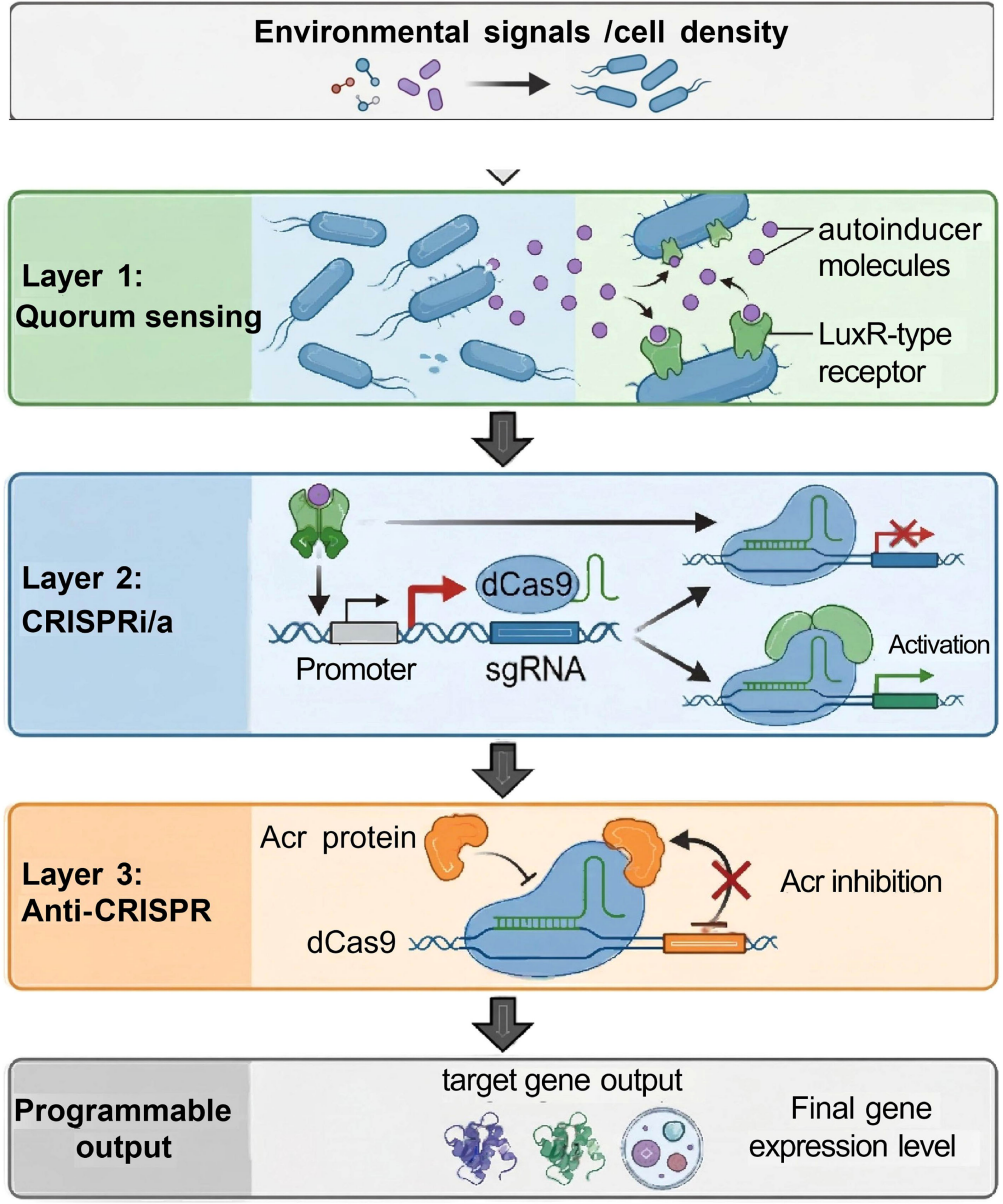
Multilayer regulatory framework integrating quorum sensing (QS), CRISPRi/a, and anti-CRISPR proteins. Environmental signals are first sensed by quorum sensing circuits that regulate gene expression in a population-dependent manner. CRISPRi/a modules provide programmable transcriptional regulation of target genes using catalytically inactive Cas proteins. Anti-CRISPR proteins act as molecular inhibitors that directly suppress Cas nuclease activity at the protein level. The integration of these regulatory layers enables hierarchical control of CRISPR systems across population, transcriptional, and protein levels, facilitating the development of adaptive and programmable genetic circuits.

Although certain binary integration systems, such as QS–CRISPR or Acr–CRISPR, have been experimentally validated, the complete integration of these three major modules into a fully functional regulatory network remains largely conceptual and lacks systematic experimental data to support such a comprehensive multi-module integration. Currently, the modular integration of quorum sensing (QS), anti-CRISPR proteins (Acr), and CRISPR interference/activation (CRISPRi/a) systems has been proposed theoretically. This framework can be abstracted into three distinct and synergistic layers(**Figure 4**): the Sensing layer, where the QS system detects changes in population density or specific environmental signals; the Regulatory layer, where CRISPRi/a enables programmable regulation of target gene expression based on these signals; and the Actuation/Inhibition layer, where Acr proteins provide rapid inhibition of CRISPR activity and negative feedback regulation.

The core idea of this layered architecture stems from the modular design principles in synthetic biology (Khalil et al., 2012), which have been validated in other single-module or binary-module systems. Specifically, the role of quorum sensing signals as input in the sensing layer (Patterson et al., 2016), the regulation of gene expression by CRISPRi/a in the regulatory layer (Qi et al., 2013), and the negative feedback regulation of Acr proteins in the execution/inhibition layer (Davidson et al., 2020) are all based on the validation of individual modules.

From the perspectives of systems biology and synthetic biology, this multi-dimensional integration strategy may potentially advance CRISPR from a single-functional molecular tool to a programmable regulatory network, enabling precise control of gene expression across temporal (dynamic response timelines), spatial (subcellular localization and compartmentalization), and population (multi-cellular cooperative behavior) dimensions. This approach provides a potential theoretical framework and engineering blueprint for the design of related systems.

## 7 Applications of multilayer CRISPR regulatory systems in bacteria

The development of CRISPR-based regulatory systems, along with modules such as Acr proteins, CRISPRi/a, and quorum sensing (QS) networks, has significantly expanded programmable gene regulation in bacteria. These systems enable precise, dynamic, and programmable control of gene expression, offering new opportunities for antimicrobial strategies and bacterial community engineering.

Quorum Sensing (QS) Modules: QS-based circuits have been applied in synthetic bacterial communities to regulate population behaviors in response to chemical signals, such as aspirin or hydrogen sulfide, enabling controlled drug release and synchronized population responses. However, QS systems as standalone antimicrobial therapeutics remain largely exploratory. Current clinical applications focus mainly on QS inhibitors, for example, targeting Pseudomonas aeruginosa infections in cystic fibrosis patients (Shaw and Wuest, 2020).

CRISPRi/a Systems: CRISPRi/a enables programmable transcriptional regulation in bacteria, facilitating the identification of essential core genes and potential antimicrobial targets (Alberts et al., 2025), as well as the optimization of metabolic pathways in industrial strains to improve product yields. CRISPR-based strategies targeting antibiotic resistance genes or virulence factors show higher specificity than conventional methods (Zhu et al., 2024).

Integration with Acr Proteins: Acr proteins provide rapid and reversible inhibition of CRISPR activity, adding an additional safety and control layer in bacterial gene regulation. Nevertheless, full integration of QS, CRISPRi/a, and Acr into unified antimicrobial circuits has been explored only in limited proof-of-concept studies. Translation of these multilayer strategies into complex *in vivo* environments remains challenging due to delivery efficiency, ecological stability, host-microbiome interactions, and potential off-target effects on symbiotic bacterial communities.

Overall, multilayer CRISPR regulatory systems hold considerable promise for programmable antimicrobial strategies in bacteria. However, their clinical and ecological applications are still at an early stage, and further research is required to overcome key technical and biological challenges.

## 8 Challenges and future prospects

This review outlines an emerging multi-layered regulatory strategy that integrates anti-CRISPR proteins (Acrs), CRISPRi/a systems, and quorum sensing (QS) mechanisms to achieve programmable gene regulation. By coupling sensing, regulation, and inhibition modules, such systems offer a conceptual framework for dynamic and context-dependent control of gene expression. While this approach holds promise in microbial engineering and synthetic biology contexts, its extension to therapeutic applications remains at an early stage and requires further validation.

### 8.1 Key challenges

Despite the potential of integrating QS, CRISPR-based regulation, and Acr proteins, multiple technical and biological challenges remain. These challenges are particularly pronounced in multi-layer systems, where interactions between modules may introduce additional complexity.

First, delivery efficiency and system compatibility represent major bottlenecks, particularly for applications in complex environments or host-associated microbiota. Existing delivery platforms, including viral and non-viral vectors, often face limitations in targeting specificity, payload capacity, and stability. In microbial systems, additional constraints such as plasmid maintenance, horizontal gene transfer, and environmental variability further complicate reliable delivery and expression.

Second, achieving precise regulatory control and predictable system dynamics remains challenging. Although Acr proteins can function as effective “off-switches” for CRISPR activity, their expression timing, dosage, and interaction with host regulatory networks require careful tuning. Improper balance between activation and inhibition may lead to signal noise, delayed responses, or incomplete repression. In multi-module systems, coupling between QS signaling and CRISPR/Acr components may also introduce nonlinear behaviors, such as response delays or unintended feedback effects.

Third, cross-species compatibility limits the transferability of these systems. Many CRISPR-Cas and Acr proteins are derived from prokaryotes and may exhibit reduced functionality when expressed in heterologous hosts. Differences in codon usage, protein folding environments, and intracellular conditions can affect expression efficiency and activity, particularly in non-native microbial or eukaryotic systems.

Finally, biosafety and ethical considerations remain critical, especially for applications involving engineered microbes or *in situ* gene regulation. Potential risks include off-target effects, unintended ecological impact, and evolutionary instability of engineered circuits. Long-term assessment of system behavior in relevant biological contexts is therefore essential.

### 8.2 Technological development directions

To address these challenges, several research directions can be pursued. Advances in computational design and machine learning have already shown utility in improving sgRNA specificity and predicting protein structure and function. These approaches can be further extended to optimize multi-layer regulatory circuits and improve system predictability. In parallel, the development of next-generation delivery strategies—including engineered bacteriophages, conjugative systems, and nanoparticle-based platforms—may enhance targeting efficiency and enable more robust gene transfer in microbial communities. Protein engineering and directed evolution provide additional opportunities to improve the stability, specificity, and compatibility of both Cas and Acr proteins. Tailoring these components for specific hosts or environmental conditions is likely to be important for expanding their applicability. Furthermore, the establishment of standardized evaluation frameworks—covering off-target effects, dynamic response profiles, and long-term stability—may facilitate comparison across studies and support the translation of these systems into practical applications.

### 8.3 Toward multi-layered regulatory systems

The integration of QS, CRISPRi/a, and Acr modules enables the construction of regulatory systems with coordinated and sequential control over gene expression. This sequential coupling suggests the potential for closed-loop-like control, where activation and deactivation are intrinsically linked through system dynamics. Compared to conventional QS–CRISPR systems, the addition of Acr-mediated inhibition provides an additional layer of temporal regulation, which may help reduce prolonged activity and potentially minimize off-target effects. However, the implementation of such systems requires careful consideration of signal thresholds, timing coordination, and component compatibility. Experimental validation in relevant microbial contexts will be necessary to assess robustness, stability, and scalability. However, such integrated systems have so far been explored primarily in simplified or engineered settings, and their performance in complex biological environments remains to be systematically evaluated.

### 8.4 Future perspectives

Looking forward, multi-module CRISPR regulatory systems may contribute to the development of more adaptive and controllable strategies in microbial engineering and biomedicine. For example, engineered microbes capable of responding to environmental cues and self-limiting their activity could potentially be applied in biosensing, metabolic engineering, or, in the longer term, therapeutic delivery.

In addition, integrating these systems with data-driven approaches may enable more precise and predictable tuning of gene expression in complex environments. However, translating such designs into real-world applications will require rigorous validation in physiologically and ecologically relevant settings, as well as careful evaluation of long-term stability and safety.

Overall, continued advances in synthetic biology, protein engineering, and systems-level modeling are expected to improve the feasibility and reliability of these integrated regulatory frameworks, supporting their broader application in microbiology and biotechnology. Despite these advances, bridging the gap between controlled experimental systems and real-world applications remains a major challenge, underscoring the need for further interdisciplinary research.
